# ARCADIA reveals spatially dependent transcriptional programs through integration of scRNA-seq and spatial proteomics

**DOI:** 10.1093/bioinformatics/btag454

**Published:** 2026-08-21

**Authors:** Bar Rozenman, Kevin Hoffer-Hawlik, Nicholas Djedjos, Elham Azizi

**Affiliations:** Department of Biomedical Engineering, Columbia University, New York, NY 10027, United States; Department of Biomedical Engineering, Columbia University, New York, NY 10027, United States; Irving Institute for Cancer Dynamics, Columbia University, New York, NY 10027, United States; Department of Computer Science, Columbia University, New York, NY 10027, United States; Department of Biomedical Engineering, Columbia University, New York, NY 10027, United States; Irving Institute for Cancer Dynamics, Columbia University, New York, NY 10027, United States; Department of Computer Science, Columbia University, New York, NY 10027, United States; Data Science Institute, Columbia University, New York, NY 10027, United States; Herbert Irving Comprehensive Cancer Center, Columbia University, New York, NY 10032, United States

## Abstract

**Motivation:**

Cellular states are strongly influenced by spatial context, but single-cell RNA sequencing (scRNA-seq) loses information about local tissue organization, while spatial proteomic assays capture limited marker panels that constrain transcriptomic inference. Integrating these modalities can elucidate how spatial niches shape transcriptional programs, yet existing approaches depend on either feature-level correspondence such as gene–protein linkage or cell-level barcode pairing, which is often unavailable.

**Results:**

We present ARCADIA (ARchetype-based Clustering and Alignment with Dual Integrative Autoencoders), a generative framework for cross-modal integration that operates without cell barcode pairing and does not assume direct feature-to-feature correspondence. ARCADIA identifies modality-specific archetypes, that is, convex combinations of cells representing extreme phenotypic states, and aligns these anchors across modalities by minimizing the discrepancy between their cell-type composition profiles. The aligned archetypes define a shared coordinate system that anchors dual variational autoencoders (VAEs) trained with cross-modal geometric regularization, preserving archetype structure and spatial neighborhood information while enabling bidirectional translation between modalities. On semi-synthetic CITE-seq data, ARCADIA outperforms existing weak-linkage methods. Applied to independent human tonsil scRNA-seq and CODEX data, ARCADIA reconstructs known tissue architecture and reveals spatially dependent transcriptional programs linking B-cell maturation and T-cell activation or exhaustion to microenvironmental niches.

**Availability and Implementation:**

Source code is accessible at https://github.com/azizilab/ARCADIA_public. Reproducibility scripts and data are available at https://github.com/azizilab/arcadia_reproducibility.

## 1 Introduction

Cell function is strongly influenced by spatial context in tissue, for example, functional subtypes that occur following intercellular communication with specific neighboring cells. Studying spatial organization and communication with transcriptomics reveals coordinated molecular networks of development, disease evolution, therapy response, and microenvironment adaptation (Ståhl *et al.* 2016, Armingol *et al.* 2021). Single-cell RNA sequencing (scRNA-seq) enables high-resolution profiling of cell phenotypes and states, yet dissociation removes spatial context (Azizi *et al.* 2018). Spatial assays including spatial transcriptomics (ST) give insight into tissue organization, but many assays are limited in unbiased discovery of gene programs and signaling, with lower throughput (e.g. limited panels in Xenium, merFISH) or resolution (e.g. multi-cell spots in Visium, Slide-seq; cross-spot diffusion in VisiumHD) and challenging segmentation that may confound single-cell assignments.

Spatial proteomics (SP, e.g. CODEX, MIBI-TOF, IMC, CosMx SMI) offer a complementary view by quantifying surface and signaling proteins, improving segmentation and communication analysis (Giesen *et al.* 2014, Goltsev *et al.* 2018, Keren *et al.* 2019, He *et al.* 2022). However, they are limited in characterizing functional impacts of communication and spatial context. Given the feasibility of collecting scRNA-seq, integration of scRNA-seq and SP benefits from the strengths of each: precise neighborhoods and interactions from SP, and fine-grained phenotypes and gene programs following interactions from scRNA-seq. Thus, there is need for robust strategies integrating scRNA-seq and SP to expose how local environment and communication shape phenotypes and composition.

eep generative models for single-cell integration (Lopez *et al.* 2018, Wu *et al.* 2021, Cao and Gao 2022, Zhao *et al.* 2022, Tang *et al.* 2024) are less suited to SP. TotalVI (Gayoso *et al.* 2021) requires paired measurements and barcodes. COVET (Haviv *et al.* 2025) uses local covariance, which is less useful for small panels. MIDAS (He *et al.* 2024) achieves mosaic integration and knowledge transfer, but relies on matching barcodes and does not use spatial information. In spatial protein and transcriptomic integration, “linkage” refers to whether features are measured in or can be predicted by both modalities (Argelaguet *et al.* 2021). Strong-linkage assumptions compress cell heterogeneity and limit transcriptome-wide inference (Stuart *et al.* 2019, Welch *et al.* 2019, Dou *et al.* 2020, 2022, Zhu *et al.* 2023). Weak-linkage approaches like MaxFuse (Chen *et al.* 2024) and scMODAL (Wang *et al.* 2025) relax feature overlap but rely on gene–protein mappings and ignore spatial context, though such context defines cell states [e.g. T-cell exhaustion in tumor microenvironments (He *et al.* 2025)].

We present ARCADIA (ARchetype-based Clustering and Alignment with Dual Integrative Autoencoders) which leverages similar cell type/state compositions between modalities and establishes correspondence by matching extreme states inferred as archetypes (He *et al.* 2025), rather than linked features. ARCADIA is designed for weakly supervised, cross-modal integration settings where harmonized cell types are available, but explicit cell and feature links are absent. ARCADIA trains separate variational autoencoders (VAEs) for scRNA-seq and SP and constrains latent spaces so that cells with similar identities co-localize in entangled embeddings. This design does not require barcode pairing, tolerates targeted panels and large feature dimensionality gaps between modalities, and explicitly respects spatial organization patterns during integration, while learning phenotypic shifts influenced by spatial context. Dual VAEs support bidirectional knowledge transfer: (i) imputation of spatial niche labels and spatially informed differential expression for scRNA-seq, and (ii) reconstruction of gene programs within and across niches for SP, informing cell communication.

We demonstrate ARCADIA on a semi-synthetic dataset of paired scRNA-seq and SP as well as the only published experimentally paired dataset of scRNA-seq and CODEX from human tonsils in separate studies. In both, ARCADIA outperforms existing methods and recapitulates spatially dependent immune states, linking modalities through composition-level priors without overfitting to sparse panels.

## 2 Materials and methods

### 2.1 Overview of ARCADIA

**Figure 1:**
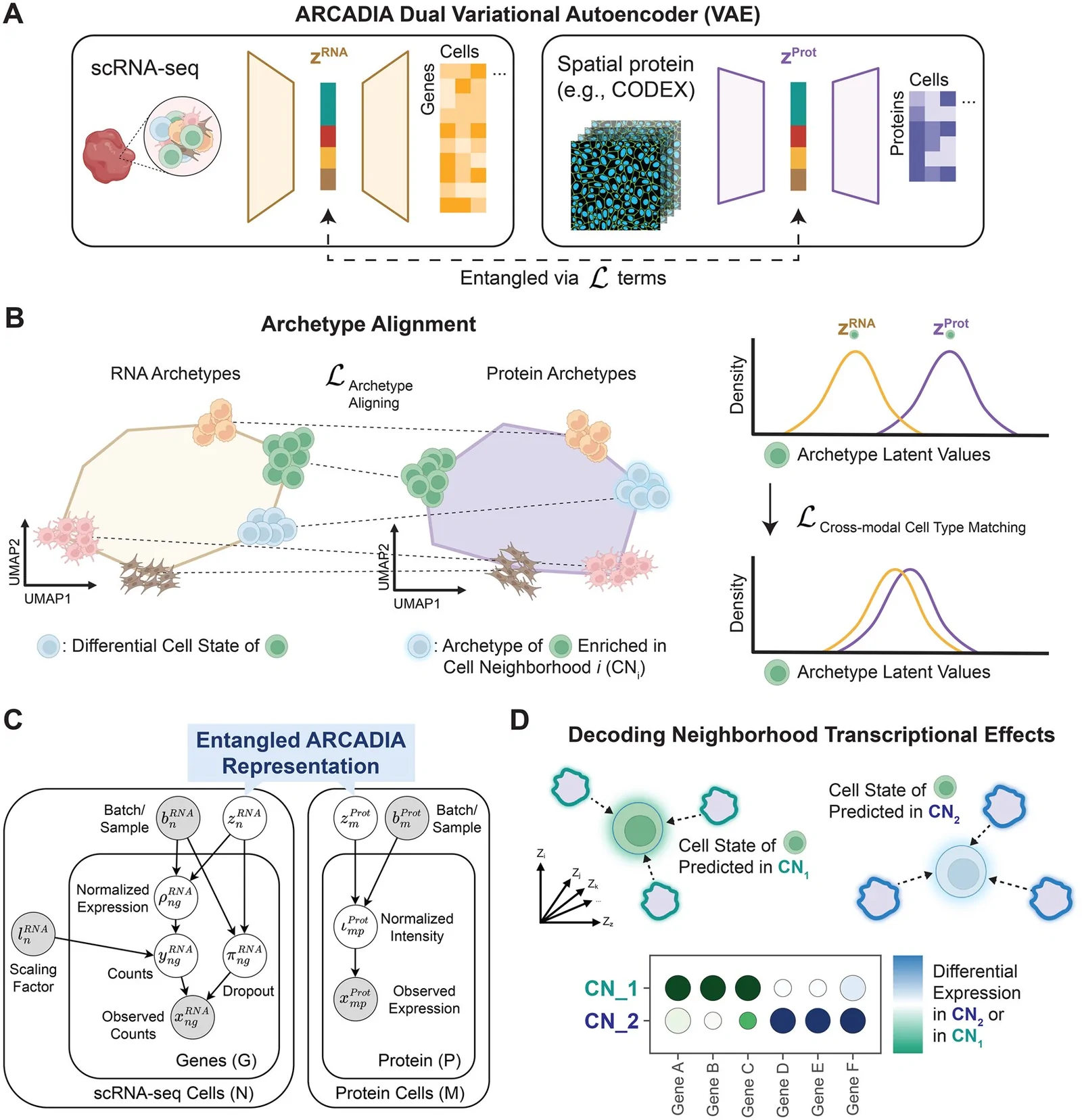
Overview of the ARCADIA framework. (A) Schematic of dual variational autoencoder (VAE) architecture including inputs and outputs. (B) ARCADIA aligns archetypes representing cell types/states in both modalities. Archetypes are convex combinations of extreme points on polytopes based on RNA expression (scRNA-seq) or self and neighborhood protein expression (spatial protein). Within matched archetypes, latent distributions are constrained using cross-modal matching loss to yield an entangled representation preserving cell type geometry and concordance across modalities. (C) Dual VAE graphical model. Latent variables are circles, and observed variables are shaded circles. (D) Data integration and trained dual VAEs allow for investigations into spatial dependencies of cell phenotypes including the effect of CNs on expression. CN, cell neighborhood.

ARCADIA employs a dual variational autoencoder (VAE) framework with coupled objectives that jointly regularize latent representations through shared biological structure inferred from expression patterns of cell types/states across modalities (Fig. 1A). Each cell is represented as a convex combination of extreme points, or “archetypes,” and latent distributions of matched archetypes are linked via cross-modal regularization to preserve geometry and encourage biological concordance (Fig. 1B). Using archetype-anchored coupling, dual VAEs learn entangled yet modality-specific representations (Fig. 1C). ARCADIA’s latent spaces enable bidirectional analysis: how phenotypic variation depends on spatial cell neighborhood and how spatial niches shape transcriptional programs (Fig. 1D).

### 2.2 Spatial protein features

For each cell, we assemble two feature types: the cell’s own protein-marker expression and spatially-derived aggregate neighborhood features. Neighborhood features are the mean expression of each protein in a spatial neighborhood (using physical coordinates). To ensure locality, long-range edges are pruned by removing connections above the 95th percentile of inter-cell distances, and the 15 nearest neighbors are retained to ensure connectivity. Given the pruned neighbor set $N_{i}$ for cell *i*, the neighborhood-mean feature for protein *p* is


$${\bar{x}}_{i,p}^{\mathrm{Prot}}=\sum_{j\in N_{i}}x_{j,p}^{\mathrm{Prot}}/|N_{i}|$$

yielding ${\bar{X}}^{\mathrm{Prot}}\in R^{M\times P}$ with *M* cells, *P* features, and rows ${\bar{x}}_{i}^{\mathrm{Prot}}=({\bar{x}}_{i,1}^{\mathrm{Prot}},\ldots ,{\bar{x}}_{i,P}^{\mathrm{Prot}})$. This matrix is z-standardized (per protein across cells), mitigating heteroscedasticity and improving numerical stability.

Neighborhood features are concatenated with protein markers to produce a spatially-aware vector. Spatial features are scaled with an adjustable hyperparameter $\alpha_{s}<1$ prior to concatenation to balance their dynamic range with that of cell-specific expression.

Cell neighborhood (CN) labels representing histological niches are obtained by K-means clustering [similar to (Ding *et al.* 2025)] of neighborhood-mean profiles. CN labels are used for evaluation of spatial separability and modeling supervision, rather than inputs.

### 2.3 Archetype analysis and alignment

ARCADIA introduces an archetype-based alignment framework that establishes biological correspondence between scRNA-seq and spatial proteomics without requiring feature-level linkage. ARCADIA learns modality-specific archetypes that capture *extreme cellular states* and aligns them using *composition-level alignment* derived from cell-type distributions. The specification of cell types for ARCADIA, as well as comparable cell-type proportions between modalities, is critical—we discuss the impact and importance of label resolution and proportion-aware subsampling in Supplementary Material.

### 2.4 Learning modality-specific archetypes

For each modality ℓ∈{RNA,Spatial Protein}, input data $X^{\left(\ell \right)}\in R^{n_{\ell }\times d_{\ell }}$, where $n_{\ell }$ denotes the number of cells and $d_{\ell }$ the number of features, is factorized by Principal Convex Hull Analysis (Cutler and Breiman 1994) to identify *k* archetypes:


$$\underset{C^{\left(\ell \right)},S^{\left(\ell \right)}}{\min}\;∥{\left(X^{\left(\ell \right)}\right)}^{⊤}-{\left(X^{\left(\ell \right)}\right)}^{⊤}C^{\left(\ell \right)}S^{\left(\ell \right)}{∥}_{F}^{2}$$

where $C^{\left(\ell \right)}\geq 0,\;S^{\left(\ell \right)}\geq 0,\;\sum_{j=1}^{n_{\ell }}C_{ji}^{\left(\ell \right)}=1,\;\sum_{i=1}^{k}S_{ij}^{\left(\ell \right)}=1$. $C^{\left(\ell \right)}\in R^{n_{\ell }\times k}$ defines archetype-selection weights, and $S^{\left(\ell \right)}\in R^{k\times n_{\ell }}$ encodes archetype mixture weights for each cell. Each archetype is a convex combination of data points:


$$A^{\left(\ell \right)}={\left(X^{\left(\ell \right)}\right)}^{⊤}C^{\left(\ell \right)}\in R^{d_{\ell }\times k},$$

and every cell is represented as a simplex-constrained mixture: ${\left(x_{i}^{\left(\ell \right)}\right)}^{T}\approx A^{\left(\ell \right)}s_{i}^{\left(\ell \right)},\;s_{i}^{\left(\ell \right)}\geq 0,\;1^{⊤}s_{i}^{\left(\ell \right)}=1,i=1,\ldots ,n_{l}$. For multi-batch data (e.g. multiple spatial proteomic samples), batch-specific archetypes are learned and then aligned.

### 2.5 Cross-modal archetype alignment

To align archetypes, we compare cell-type composition signatures. Given cell-type set T in each modality ℓ, we compute archetype-by-type proportion matrices $\Phi^{\left(\ell \right)}\in R^{k\times |T|}$:


$$\Phi^{\left(\ell \right)}[i,t]=\frac{\left|\{j\in G_{i}^{\left(\ell \right)}:c_{j}=t\}\right|}{\left|G_{i}^{\left(\ell \right)}\right|}.$$

$G_{i}^{\left(\ell \right)}=\{j:\arg\max_{r}S_{rj}^{\left(\ell \right)}=i\}$ is the set of cells assigned to archetype *i*, based on the highest archetype mixture weight (see Supplementary Material), and $c_{j}$ is the cell-type of cell *j*. We then find the optimal permutation $\pi^{*}$ that aligns archetypes across modalities by minimizing the cosine distance between their composition profiles:


$$\pi^{*}=\arg\underset{\pi \in S_{k}}{\min}\sum_{i=1}^{k}d_{\text{cosine}}(\Phi^{\left(\text{RNA}\right)}[i,:],\Phi^{\left(\text{Prot}\right)}[\pi (i),:])$$

where $S_{k}$ is the set of all permutations of *k* elements. We solve this optimization using the Hungarian method in $O(k^{3})$ time (Kuhn 1955). The optimal $k^{*}$ minimizes the average alignment cost: $k^{*}=\arg\min_{k}\frac{1}{k}\sum_{i=1}^{k}d_{\text{cosine}}(\Phi^{\left(\text{RNA}\right)}[i,:],\Phi^{\left(\text{Prot}\right)}[\pi^{*}(i),:])$.

### 2.6 Extreme cell-state anchors

Cells whose archetype mixture vectors $s_{i}^{\left(\ell \right)}$ are dominated by a single archetype are denoted *anchors* and used as high-confidence archetypes for modality alignment. Anchors represent biologically distinct cell states enabling robust archetype-level alignment prior to latent-space training.

In contrast to feature-level alignment methods, ARCADIA defines cross-modal correspondence via archetype matching, establishing interpretable links between transcriptional and spatial cell states as the biologically grounded foundation for dual-VAE training. Further details on archetype analysis and cross-modal alignment are in the Supplementary Material.

### 2.7 Model architecture and training

Both the RNA and protein encoders project to respective 60-dimensional latent spaces. This larger dimensionality compared to (Chen *et al.* 2024, Wang *et al.* 2025) reflects high-dimensional inputs: highly variable genes without filtering to protein markers for RNA, and markers concatenated with neighborhood features for protein. Both encoders employ batch normalization and dropout (ρ=0.1), and decoders mirror each encoder. A single AdamW optimizer handles trainable parameters, updating both VAEs in each pass.

For RNA we use a zero-inflated negative binomial (ZINB) likelihood on counts. For protein we use a Gaussian likelihood. We select likelihoods by fitting candidates to random samples from each modality and evaluating Akaike Information Criterion (AIC). Batch labels condition decoders during training, allowing VAEs to absorb batch effects in the latent space. Combined with per-batch archetype alignment, this yields consistent representations across batches and modalities.

As VAEs encode/decode independently, trained VAEs support cross-modal inference [similar to generating counter-factuals in Boyeau *et al.* (2025)]. Additional model details (e.g. generative and inference processes) are in Supplementary Material.

### 2.8 Objective functions

Dual VAEs learn latent spaces by optimizing four objectives. Modality-specific evidence lower bounds (ELBOs) ensure faithful feature reconstruction while regularizing latent geometry. Structure-preservation loss maintains cell-type relationships and organization so latent geometry reflects known biological structure in both modalities. Cross-modal Maximum Mean Discrepancy (MMD) loss encourages mixing of cell types, promoting correspondence without feature-level alignment. Anchor-guided loss preserves intra-cell-type variation, preventing over-mixing and ensuring phenotypic diversity within cell types is retained. Together, the loss terms entangle latent spaces while enabling cross-modal inference.

### 2.9 Evidence lower bound (ELBO)

For modality ℓ∈{RNA,Spatial Protein}, we define the ELBO:


$$\begin{array}{cc} L_{\text{ELBO}}^{\left(\ell \right)}=-E_{q_{\phi }(z^{\left(\ell \right)}|x^{\left(\ell \right)})}[\text{log}\;p_{\theta }(x^{\left(\ell \right)}|z^{\left(\ell \right)})] & \\ \;+\text{KL}(q_{\phi }(z^{\left(\ell \right)}|x^{\left(\ell \right)})∥p(z)). & \end{array}$$

$x^{\left(\ell \right)}$ is observed data, $z^{\left(\ell \right)}$ is the latent representation, $q_{\phi }$ is the variational encoder, and $p_{\theta }$ is the decoder. We employ standard Kullback–Leibler divergence with normal priors p(z)=N(0,I).

### 2.10 Anchor-guided cross-modal matching

*Anchors* are cells whose archetype vectors $s_{i}^{\left(\ell \right)}$ are dominated by a single archetype, so they serve as high-confidence alignment references. The matching loss penalizes distances between anchors via softplus with adaptive margins:


$$\pi^{*}=\arg\min_{\pi \in S_{k}}\sum_{i=1}^{k}d_{\text{cosine}}(\Phi^{\left(\text{RNA}\right)}[i,:],\Phi^{\left(\text{Prot}\right)}[\pi (i),:])$$

where E is the set of anchors in each modality ℓ, $d_{i}^{\text{close}}$ and $d_{i}^{\text{far}}$ are latent-space distances from anchor *i* to its most and least similar counterpart in the other modality, $m_{\text{close}}=\alpha_{\text{close}}\sigma$ is a closeness margin ($0<\alpha_{\text{close}}\ll 1$), and $\tau_{\text{far}}=\alpha_{\text{far}}\sigma$ is a farness margin ($\alpha_{\text{far}}>1$), with σ as the standard deviation of latent distances. $\mathrm{softplus}(x)=\frac{1}{\beta }\text{log}(1+e^{\beta x})$ provides smooth, non-negative penalties. Critically, when distances fall within acceptable thresholds (i.e. close enough or far enough), the penalty is near zero, so well-matched pairs contribute minimally while poorly-matched pairs incur larger penalties. This design encourages natural alignment without hard constraints.

### 2.11 Cell-type structure preservation

For each modality and each cell type t∈T, cell-type centroids are computed in archetype space and VAE latent space. Structure preservation loss aligns inter-cell-type affinity matrices computed via Gaussian kernels:


$$L_{\text{struct}}^{\left(\ell \right)}=\frac{1}{\left|T|(|T|-1\right)}\sum_{i\neq j}{\left(W_{ij}^{\left(\ell \right)}-{\tilde{W}}_{ij}^{\left(\ell \right)}\right)}^{2}$$

where $W_{ij}^{\left(\ell \right)}=\text{exp}(-\frac{∥\mu_{i}^{\left(\ell \right)}-\mu_{j}^{\left(\ell \right)}{∥}_{2}^{2}}{2\sigma_{\ell }^{2}})$ is the affinity between cell types *i* and *j* in latent space with centroids $\mu_{t}^{\left(\ell \right)}$, and ${\tilde{W}}_{ij}^{\left(\ell \right)}$ is the corresponding affinity in archetype space with centroids ${\tilde{\mu }}_{t}^{\left(\ell \right)}$. Additional cohesion and separation losses promote cluster compactness and inter-cluster distinctness (see Supplementary Material).

### 2.12 Cross-modal alignment

We compute the Maximum Mean Discrepancy (MMD) between distributions of cell types in each latent $z^{\left(\ell \right)}$ to align them. Minimizing MMD ensures cell types occupy similar regions in $z^{\left(\ell \right)}$ while facilitating mixing and preventing modality-specific artifacts. For each cell type t∈T, $Z_{t}^{\left(\ell \right)}\in R^{N_{t}^{\left(\ell \right)}\times q}$ denotes the matrix of latent representations in modality ℓ, where $N_{t}^{\left(\ell \right)}$ is the number of type-*t* cells and *q* is the latent dimension. Each row of $Z_{t}^{\left(\ell \right)}$ is a cell’s latent embedding. The MMD is:


$$\begin{array}{cc} {\text{MMD}}_{t}^{2}=\frac{1}{N_{t}^{\left(\text{RNA}\right)}(N_{t}^{\left(\text{RNA}\right)}-1)}\sum_{i\neq j}k(z_{i}^{\left(\text{RNA}\right)},z_{j}^{\left(\text{RNA}\right)})\\ & \;+\frac{1}{N_{t}^{\left(\text{Prot}\right)}(N_{t}^{\left(\text{Prot}\right)}-1)}\sum_{i\neq j}k(z_{i}^{\left(\text{Prot}\right)},z_{j}^{\left(\text{Prot}\right)})\\ & \;-\frac{2}{N_{t}^{\left(\text{RNA}\right)}N_{t}^{\left(\text{Prot}\right)}}\sum_{i,j}k(z_{i}^{\left(\text{RNA}\right)},z_{j}^{\left(\text{Prot}\right)}) \end{array}$$

where the RBF kernel is $k(x,y)=\text{exp}\;\left(-∥x-y{∥}_{2}^{2}/2\sigma^{2}\right)$ and σ is adaptively set via the median heuristic on combined data. Specifically, $\sigma_{m}=\text{median}\{∥x-y{∥}_{2}:x\in Z_{t}^{\left(\text{RNA}\right)},y\in Z_{t}^{\left(\text{Prot}\right)}\}$ scales the kernel automatically to the data’s natural distance distribution. Instead of a single bandwidth, an ensemble of MMDs (Schrab *et al.* 2023) is computed at multiple scales: fine- ($\sigma_{m}^{\text{fine}}=\sigma_{m}/2$), medium- ($\sigma_{m}^{\text{med}}=\sigma_{m}$), and coarse-grained ($\sigma_{m}^{\text{coarse}}=2\sigma_{m}$):


$$\begin{array}{cc} {\text{MMD}}_{t,\text{ensemble}}^{2}=\frac{1}{\left|\Sigma_{\text{scales}}\right|}[{\text{MMD}}_{t}^{2}(\sigma_{m}^{\text{fine}}) & \\ \;+{\text{MMD}}_{t}^{2}(\sigma_{m}^{\text{med}})+{\text{MMD}}_{t}^{2}(\sigma_{m}^{\text{coarse}})]. & \end{array}$$

Fine-grained scales detect tightly clustered cell types, medium scales capture intermediate structure, and coarse-grained scales capture loosely distributed ones, ensuring alignment respects differences in how cell types disperse in the latent space. Cross-modal alignment loss is the average MMD across cell types:


$$L_{\begin{array}{c} \text{cross}-\\ \text{modal} \end{array}}=\frac{1}{\left|T_{\text{common}}\right|}\sum_{t\in T_{\text{common}}}{\text{MMD}}_{t,\text{ensemble}}^{2}$$

MMD is calculated on batches with balanced counts of RNA and protein cells, to avoid imbalances affecting MMD.

### 2.13 Total training objective

The total loss combines components via fixed hyper-parameters:


$$L_{\text{total}}=\sum_{\ell }\left[\lambda_{0}^{\left(\ell \right)}L_{\text{ELBO}}^{\left(\ell \right)}+\lambda_{1}^{\left(\ell \right)}L_{\text{struct}}^{\left(\ell \right)}\right]+\lambda_{2}L_{\begin{array}{c} \text{cross}-\\ \text{modal} \end{array}}+\lambda_{3}L_{\text{anchors}}\;$$

where $\lambda_{0}^{\left(\ell \right)}$ and $\lambda_{1}^{\left(\ell \right)}$ control reconstruction and structure preservation (including additional cohesion and separation losses) and $\lambda_{2}$ and $\lambda_{3}$ control alignment strength. Further details on objective functions are in Supplementary Material.

## 3 Experiments and results

### 3.1 Datasets

#### 3.1.1 Semi-synthetic dataset

**Figure 2:**
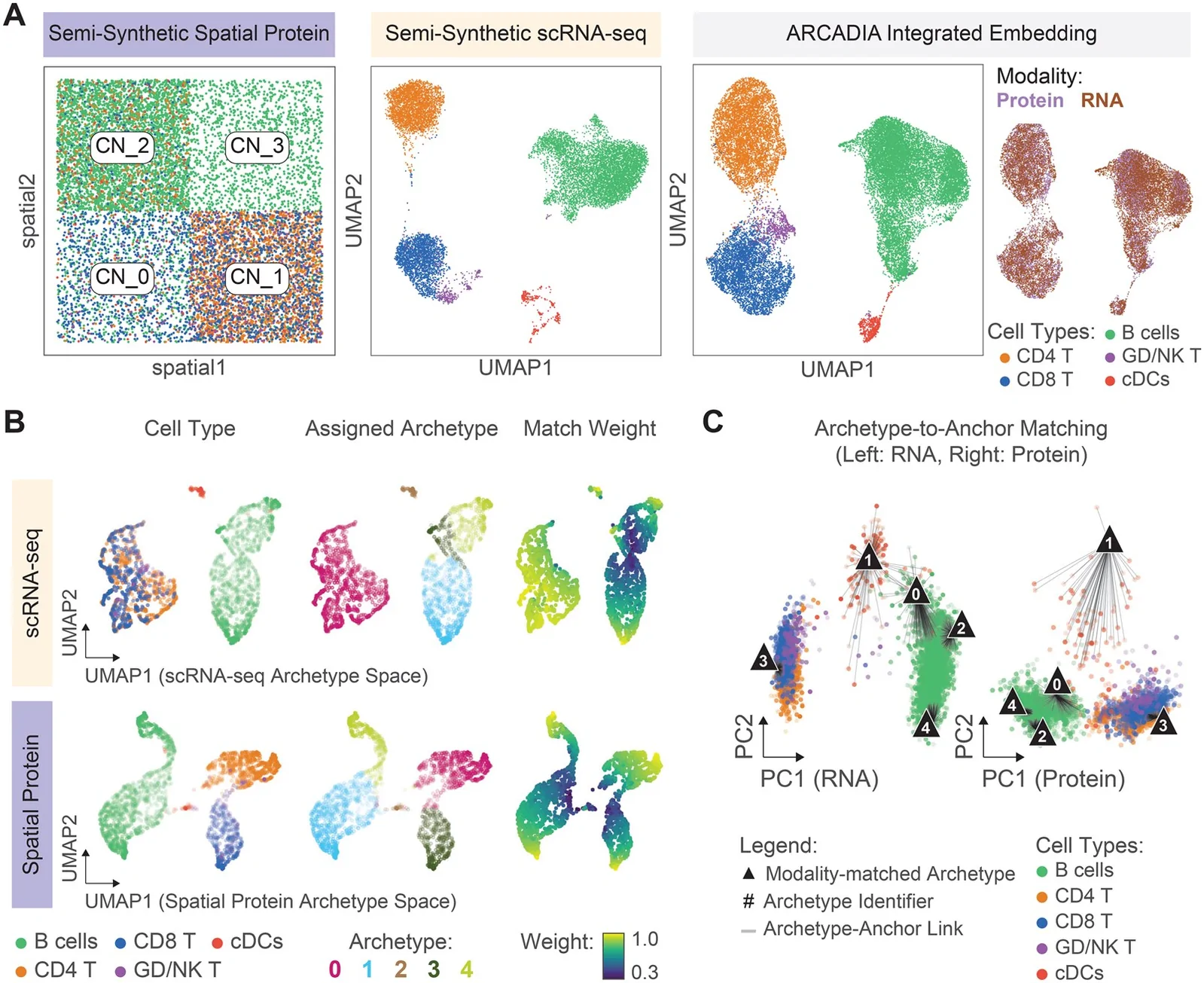
Semi-synthetic data integration and archetype alignment. (A) Left, spatial grid of protein cells and UMAP of RNA cells colored by type; right, ARCADIA embedding colored by type (outer) and modality (inner). (B) RNA and protein cells in archetype space. Opacity reflects match weight. (C) Archetype matching for RNA and protein cells in PC space. CN, cell neighborhood.

To define ground-truth spatial niches dictating cell states, we took an expertly annotated CITE-seq dataset with paired protein and RNA expression (Hao *et al.* 2021), split it into two disjoint sets of protein- and RNA-expressing cells, and positioned protein cells on a spatial grid where each quadrant contained certain subtypes. Specifically, we placed different B cells subtypes in cell neighborhoods (CNs), surrounded either by no other cell types or by T and dendritic cells. We held out spatial context information for RNA cells. Protein profiles alone could not describe the range of B cell states, but could be linked to RNA profiles to describe these states. As such, we tested whether ARCADIA learns cross-modal cell types based on aligned archetypes as well as spatially-dependent variation (Fig. 2A).

#### 3.1.2 Human tonsil dataset

To demonstrate the utility of ARCADIA to discover spatially-dependent cell state patterns, we integrated human tonsil data collected in separate studies (Kennedy-Darling *et al.* 2021, King *et al.* 2021a, 2021b). At the time of this study, this collection is the only publicly available, experimentally-paired scRNA-seq and CODEX dataset. Both modalities were already expertly-annotated. As in Ding *et al.* (2025), we characterized 10 cell neighborhoods (CNs) based on protein expression of each cell and its surrounding neighbors.

## 4 Data preprocessing

We performed standard quality control (low-quality RNA cell/gene removal, median absolute deviation outlier removal for protein data), feature selection (highly variable genes for scRNA-seq), and normalization (library size scaling and log transform for scRNA-seq, per-protein z-scoring) steps. We also harmonized cell type labels, which is crucial for archetype alignment. Preprocessing protocols, including dataset-specific considerations, are in Supplementary Material.

### 4.1 Computational benchmarking

We assessed ARCADIA’s scalability using the semi-synthetic CITE-seq dataset (∼30k total cells) and the human tonsil dataset (∼180k total cells). Preprocessing runtime, including spatial graph construction and archetype generation, scaled with dataset size. In contrast, dual-VAE training (300 epochs) was comparable (1.3 vs. 1.8 hours), as cost is driven by model complexity. Crucially, peak GPU memory usage was low for both datasets (3.0–3.2 GB), demonstrating ARCADIA can process large spatial datasets on standard hardware (NVIDIA T4) (Table 1). Detailed hardware specifications, complexity analysis, and resource profiling are in the Supplementary Material.

**Table 1:** Dataset statistics and computational benchmarks.

Metric	CITE-seq	Tonsil
Dimensions (cells × genes or features)		
scRNA-seq	15 337 × 1796	12 917 × 2000
Spatial Protein	14 021 × 220	169 468 × 92
Preprocessing (CPU)		
Runtime	13.5 min	56.9 min
Peak System RAM	14.9 GB	38.6 GB
Training (GPU, 300 Epochs)		
Trainable Parameters	∼ 15.3 Million	∼ 16.0 Million
Runtime	1.3 h	1.8 h
Peak System RAM	7.7 GB	41.7 GB
Peak GPU VRAM	3.0 GB	3.2 GB
Avg. CPU Usage	197% (∼2 cores)	190% (∼2 cores)

Benchmarks using 8-core Intel Xeon CPU and 15 GB VRAM NVIDIA Tesla T4 GPU. For additional details, see Supplementary Material.

### 4.2 Performance on semi-synthetic dataset

ARCADIA exhibited robust and meaningful archetype matching in both the RNA and protein expression space (Fig. 2B and C) and clear separations by cell type with even distribution of cells across modalities in the integrated embedding space (Fig. 2A, Supplementary Fig. S1A). Crucially, beyond co-embedding immune populations, ARCADIA captured the spatial patterning of B-cell subtypes across CNs (Supplementary Fig. S2A and B), evidenced by clear cell clusters from different CNs in the integrated embedding (Supplementary Fig. S2C). MaxFuse (Chen *et al.* 2024) and scMODAL (Wang *et al.* 2025) integrated cell types, but could not learn spatially-dependent cell state patterns (Supplementary Fig. S2C).

**Figure 3:**
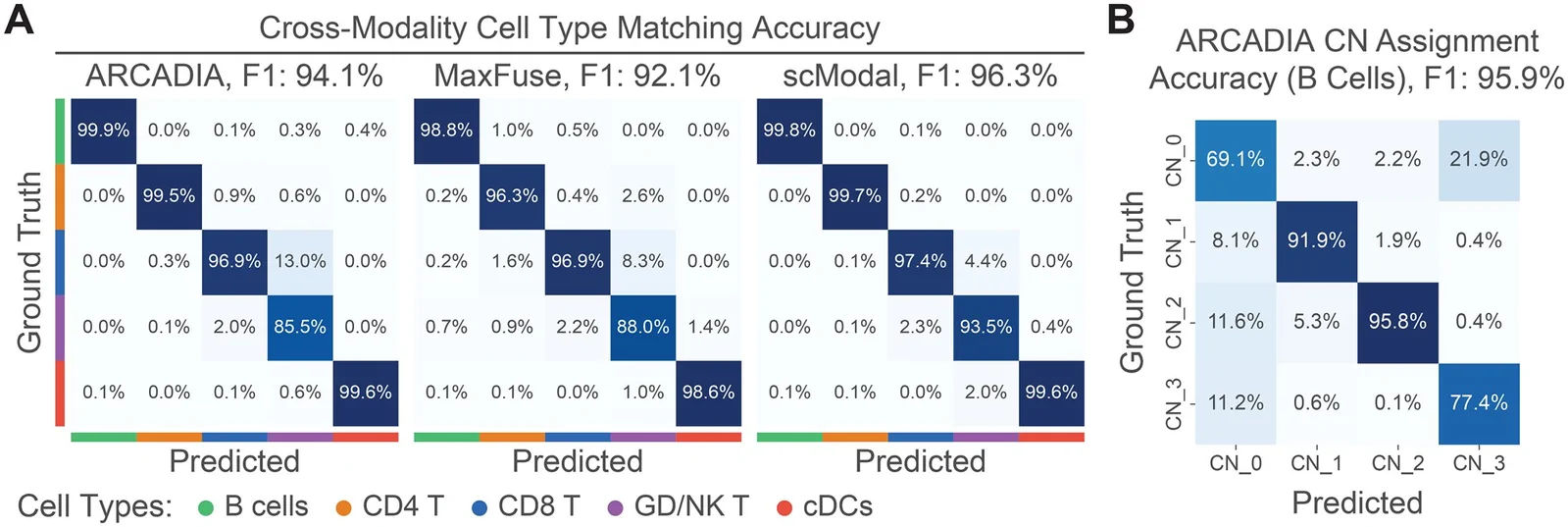
Integration performance on semi-synthetic data. (A) Cell type confusion matrices for ARCADIA, MaxFuse, and scMODAL. (B) Confusion matrices for B cell-specific CN prediction from ARCADIA.

ARCADIA out-performed both MaxFuse and scMODAL in most benchmarking metrics, while all three comparably predicted cell types (Table 2, Fig. 3A). For each method we used integrated embeddings to predict CNs from scRNA-seq by assigning each RNA cell its probable CN based on the 15 nearest protein neighbors, and compared them to ground-truth, to test how each method could recover cell subtypes from spatial grid location (in protein) and phenotype (in RNA). ARCADIA significantly outperformed both MaxFuse and scMODAL using CN F1 scores (Table 2). In particular, ARCADIA accurately predicted spatial location for B cell subtypes in CN_1 and CN_2, which had the greatest density and subtype diversity (Fig. 3B, Supplementary Fig. S2A and B). See Table S1 in Supplementary Material for additional benchmarking.

**Table 2:** Dataset integration performance benchmarking.

	**Semi-synthetic CITE-seq dataset**			**Tonsil dataset**		
Metric	ARCADIA	MaxFuse	scMODAL	ARCADIA	MaxFuse	scMODAL
kSep	**0.666** ± **0.046**	0.403	0.352	**0.510** ± **0.016**	0.212	0.449
CN F1	**0.979** ± **0.003**	0.556	0.577	–	–	–
Cell-type F1	0.941 ± 0.011	0.921	**0.963**	**0.897** ± **0.003**	0.888	0.848
ARI Score	0.972 ± 0.008	0.955	**0.991**	**0.791** ± **0.011**	0.680	0.635
iLISI	**1.771** ± **0.050**	1.704	1.600	1.543 ± 0.038	1.208	**1.690**
Silhouette Score	**0.607** ± **0.028**	0.086	0.301	**0.315** ± **0.017**	0.061	0.159

Bold indicates best performance. ARCADIA scores are mean ± std across 10 runs. *kSep*: k-nearest neighbor separation test measuring CN distinctiveness within cell-type clusters (higher indicates better separation) (Büttner *et al.* 2019); CN/Cell-type F1, F1 accuracy score across modalities; ARI, Accuracy between predicted and ground-truth labels; iLISI, Modality mixing in local neighborhoods (max = 2) (Korsunsky *et al.* 2019); Silhouette, Latent space cluster separation quality. For additional details, see Supplementary Material.

### 4.3 Performance on human tonsil dataset

**Figure 4:**
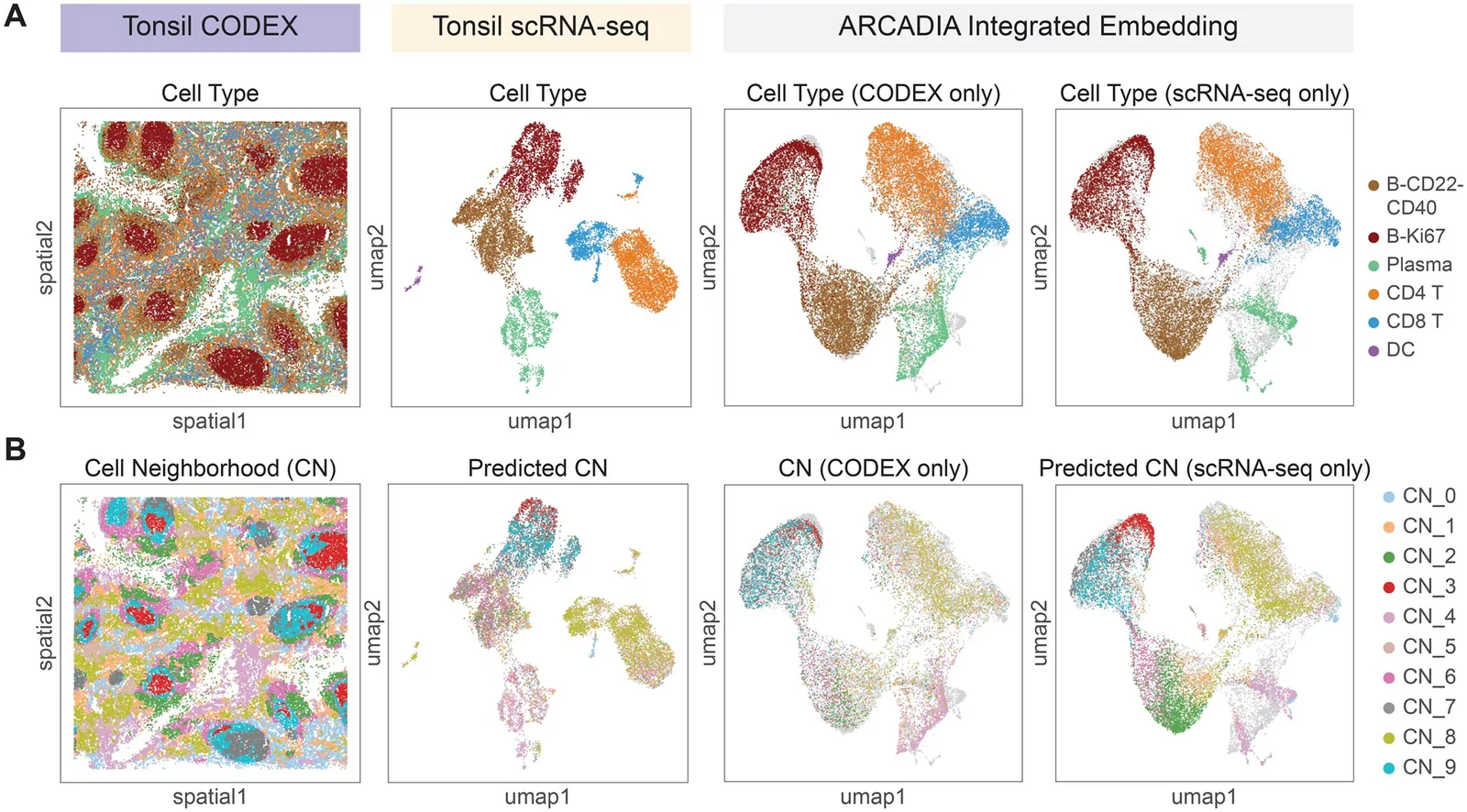
ARCADIA integrates human tonsil scRNA-seq and CODEX data. (A) Cell types in the CODEX tonsil tissue sample (left), in scRNA-seq (middle), and in ARCADIA space (right). (B) Computed cell neighborhoods (CNs) in the CODEX tissue sample (left), predicted CN from scRNA-seq (middle), and ground-truth and predicted CN in ARCADIA space (right). DC, dendritic cell.

Next we studied spatially-dependent behavior of immune cells. ARCADIA recovered major types (Fig. 4A) and cell-state gradients across spatial contexts (Fig. 4B, Supplementary Fig. S3A). While MaxFuse and scMODAL were comparable in broad cell type integration (Supplementary Fig. S3B, Table 2), only ARCADIA captured spatially-dependent B, T, and dendritic cell states (Supplementary Fig. S3C).

Previous studies characterized spatial structures of germinal-centers and peripheral regions in the tonsil (Ding *et al.* 2025). ARCADIA’s embedding links spatial substructures (CNs) to the spectrum of states of B, T, and dendritic cells. In predicting CN for each cell, ARCADIA allows for spatially-informed transcriptomic analysis at the level of cells in CNs, as opposed to the level of CNs (Ding *et al.* 2025). We next used the ARCADIA framework to contrast states of B, CD8 T, and CD4 T cells within different spatial regions.

### 4.4 ARCADIA learns spatially-resolved gene expression patterns

We used ARCADIA to predict CN membership for each scRNA-seq cell and calculated differentially expressed genes (DEGs) within cell types but across inferred CNs, unbiasedly studying how phenotype varies by tonsil CN. DEGs were restricted to CNs with adequate cell type representation (Supplementary Fig. S3A). Complete lists of top DEGs are in Supplementary Figs. S4, S5, and S6.

**Figure 5:**
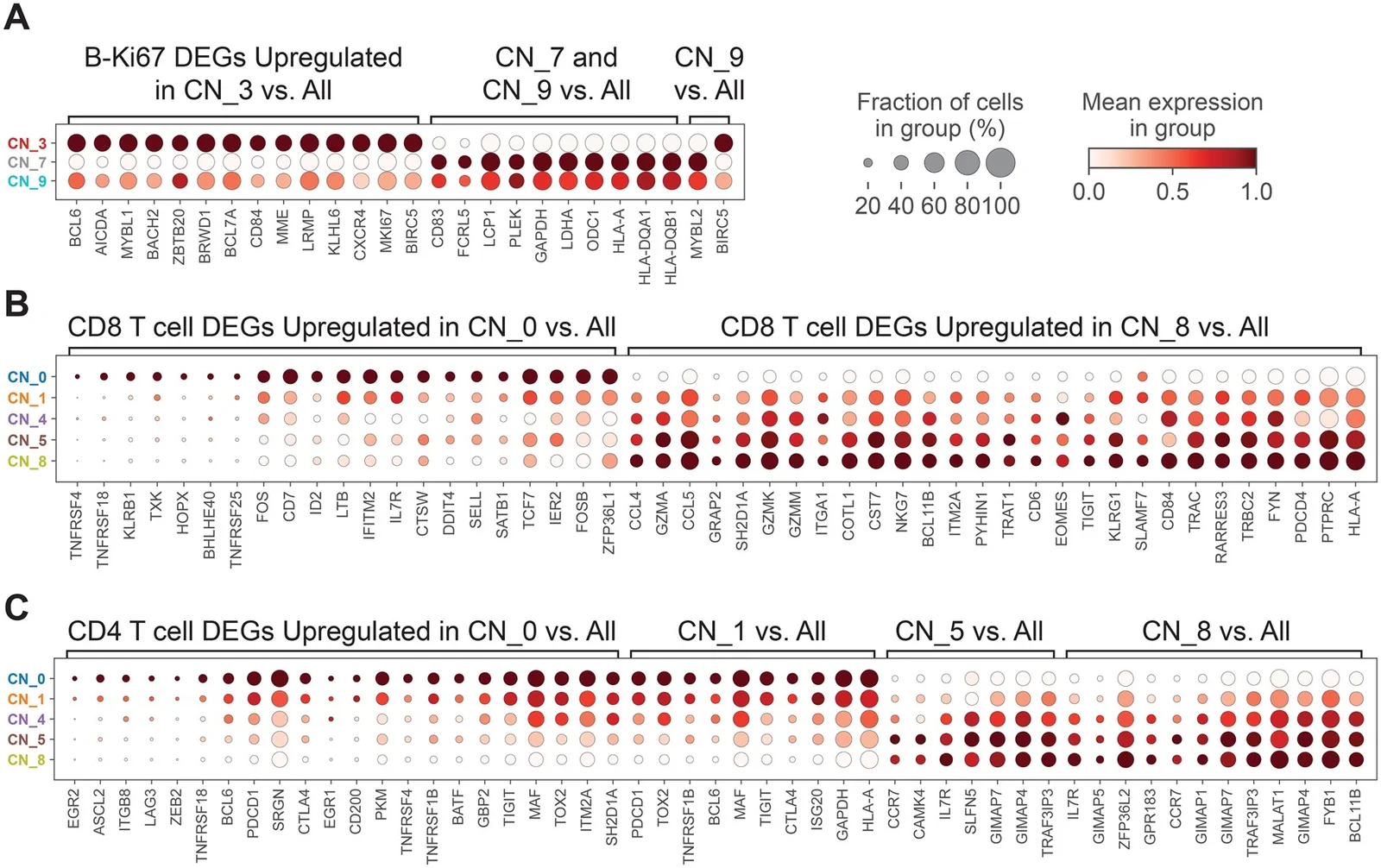
Spatially-resolved differential expression. Genes expressed by B (A), CD8 T (B), and CD4 T cells (C) predicted to originate from specific CNs. Only CNs with sufficient representation are shown (see Supplementary Fig. S3A). Expression is scaled across CNs. Full DEGs in Supplementary Figs. S4–S6.

B cells in central germinal-center (GC) CN_3 expressed hypermutation (*BCL6, AICDA, MYBL1, BACH2*), activation/BCR signaling (*CD84, MME, LRMP, CXCR4*), and proliferation markers (*MKI67, BIRC5*). Peripheral CN_7 and CN_9 B cells expressed plasma-cell-like (*CD83, FCRL5*), metabolic (*GAPDH, LDHA, ODC1*), and antigen-presentation (*HLA-A, HLA-DQA1, HLA-DQB1*) genes (Fig. 5A). These results suggest B cells deep within GCs proliferate/hypermutate, whereas in outer GC zones they undergo terminal differentiation and immune-surveillance.

CD8 T cells in CN_0 had effector (*TNFRSF4, CTSW*), activated (FOS, LTB), and stem-like (*IL7R, TCF7*) states, while those in CN_8 co-expressed cytotoxic (*CCL4, GZMA*) and exhaustion (*TIGIT, KLRG1, PDCD4*) markers (Fig. 5B). CD4 T cells in CN_4 showed terminally exhausted phenotypes (*ZEB2, LAG3, CTLA4*), whereas CN_1 displayed T follicular helper (Tfh)-like states (*BCL6, MAF, GAPDH, HLA-A*) and CN_5/8 contained stem-like precursors (*IL7R, CCR7*) (Fig. 5C). Additional DEGs and discussion are in Supplementary Material.

Thus, ARCADIA reveals coordinated B-cell maturation and T-cell activation/exhaustion dependent on spatial niche relative to GCs, recapitulating canonical B and Tfh cell biology (Crotty 2011, De Silva and Klein 2015) and understudied follicular CD8 T-cell behaviors (Koh *et al.* 2023).

## 5 Discussion and future work

As additional low-to-medium feature, single-cell spatial omics datasets become available with paired scRNA-seq, ARCADIA provides a unified framework to probe relationships between tissue microenvironment and cell state, including in weak-linkage multimodal settings where archetype alignment is still useful. Benchmarks on multiple datasets show that ARCADIA outperforms weak-linkage methods and uniquely recovers spatially-dependent functional states across immune lineages. We note model accuracy depends on balanced cell-type representation across data, since archetype alignment relies on shared composition, and that substantial imbalances in feature dimensionality or cell counts between modalities may require iterative levels of feature selection or multi-batch subsampling to avoid biases during archetype construction or VAE training (e.g. MMD). In particular, feature dimensionality gaps are critical to consider to ensure mutually apparent archetypes, although ARCADIA is designed to absorb feature asymmetries via archetype-linked VAEs. In the case of studies without perfectly matched samples, ARCADIA can still provide useful integration of mutually-apparent archetypes. As with other deep generative models, careful hyperparameter tuning is required to maintain biologically meaningful separation of subtle states. Future work will extend cross-modal decoding (e.g. predicting RNA expression from CODEX). In sum, ARCADIA establishes a generative, interpretable bridge between scRNA-seq and SP and bidirectional modeling of interactions and tissue remodeling.

## Supplementary Material

btag454_Supplementary_Data

## Data Availability

All data analyzed are previously published. Source code is accessible at https://github.com/azizilab/ARCADIA_public. Reproducibility scripts and data are available at https://github.com/azizilab/arcadia_reproducibility.
